# Preparation, Characterization, and Biological Activities of Topical Anti-Aging Ingredients in a *Citrus junos* Callus Extract

**DOI:** 10.3390/molecules22122198

**Published:** 2017-12-11

**Authors:** Deepak Adhikari, Vijay Kumar Panthi, Rudra Pangeni, Hyun Jung Kim, Jin Woo Park

**Affiliations:** College of Pharmacy and Natural Medicine Research Institute, Mokpo National University, Muan-gun, Jeonnam 58554, Korea; dpak7adh@gmail.com (D.A.); nepalivijay7@gmail.com (V.K.P.); capriconpangeni@gmail.com (R.P.)

**Keywords:** anti-aging, callus extract, cosmetic ingredients, *Citrus junos*, plant cell culture, skin lightening

## Abstract

In this study, we prepared and characterized a callus extract from *Citrus junos* and assessed its utility as a source of topical anti-aging ingredients. Callus extract was produced by aqueous extraction from *Citrus junos* grown on Murashige and Skoog medium with picloram as a growth regulator. After measuring the total phenolic and flavonoid contents, the major phenolic compound in calli was identified as *p*-hydroxycinnamoylmalic acid (**1**) by spectroscopic analysis. The total phenol content in the extract was determined to be 24.50 ± 0.43 mg/g of gallic acid equivalents; however, the total flavonoid content of the extract was not determined. The biological activities of the callus extract, in terms of skin anti-aging, were assessed by measuring the anti-tyrosinase activity in, and melanogenesis by, melanoma cells; and proliferation of, and procollagen synthesis by, human fibroblasts. The callus extract was incorporated into nanoliposomes (NLs) to improve its percutaneous absorption. Addition of the callus extract resulted in a 1.85-fold decrease in the melanin content of melanocytes compared with that with arbutin. The extract (500 μg/mL) significantly promoted the proliferation of, and procollagen synthesis by, fibroblasts (by 154% and 176%, respectively). In addition, the flux through the human epidermis of *Citrus junos* callus extract incorporated into NLs was 17.67-fold higher than that of the callus extract alone. These findings suggest that *Citrus junos* callus extract-loaded NLs have promise as an anti-aging cosmetic, as well as having a skin-lightening effect.

## 1. Introduction

Plant tissue culture is a valid alternative method for production of pharmaceutical and cosmeceutical ingredients; fresh material is always available regardless of the season or the plant reproductive cycle [1,2]. In addition, growing conditions are readily standardized, affording high-level consistency from batch to batch, and the extracted components are safe and pure; there is no risk of pathogenic or environmental contamination. Genetic or biochemical modifications may increase the concentrations of desired bioactive compounds [3,4].

*Citrus junos* Siebold ex. Tanaka (Rutaceae), also known as *yuja* in Korea, is a citrus tree with edible yellow fruits that has been reported to inhibit oxidative stress and inflammation [5]. In previous studies, several active compounds, such as rutin, quercetin, tangeretin, naringin, and hesperidin were found in the fruit; such anti-oxidants prevent or retard the oxidation of other molecules by interrupting the initiation or propagation of oxidizing chain reactions [6,7]. In addition, such metabolites exhibit significant protective biological activities, including anticancer, anti-inflammatory, and antiviral properties [8,9]. Furthermore, citrus peel extract is an effective anti-oxidant, anti-acne, and skin-whitening agent, as it is very rich in phenolic compounds including phenolic acids and flavonoids [10,11].

Phenolic compounds are widely accepted as effective scavengers of reactive oxygen species (ROS) like singlet oxygen, the superoxide anion radical, the hydroxyl radical, and hydrogen peroxide [12]. Previous studies found that persistent exposure to ROS triggered skin aging via destruction of the anti-oxidant system, wrinkle formation, and melanogenesis [13,14]. Free radicals can trigger the formation of collagen cross-links, causing the skin to lose elasticity and thus initiating aging [15]. However, phenolic compounds serve as reducing agents, hydrogen donors, and singlet oxygen quenchers. These qualities render them extremely useful in cosmetic formulations that seek to prevent or retard oxidative damage to collagen caused by free radicals. Therefore, antioxidants containing a variety of bioactive components from natural sources have been used to prevent aging [16].

The physicochemical profiles of promising bioactive compounds must be considered in terms of successful transport of the materials to the desired site of action across the barrier of the stratum corneum (SC). Thus, many studies have tried to improve the potency, solubility, and skin permeability of selected compounds, or to encapsulate them in colloidal nanocarriers, such as solid lipid nanoparticles, nanostructured lipid carriers, nanoemulsions, nanoparticle suspensions, and polymeric nanoparticles, to facilitate skin permeation and protect against degradation [17,18,19].

In this study, we focused on the preparation and characterization of a callus extract from *Citrus junos* and assessed its 2,2-diphenyl-2-picrylhydrazyl (DPPH) free radical-scavenging activity. Next, we evaluated the skin-lightening and regeneration efficacies of the extract by measuring its capacity to inhibit tyrosinase activity and melanin biosynthesis, and to stimulate the proliferation of, and procollagen synthesis by, human fibroblasts; no report on the biological utility of *Citrus junos* callus extract, in terms of skin anti-aging, has yet appeared. Finally, the callus extract was incorporated into nanoliposomes (NLs) and delivered topically to enhance percutaneous absorption and improve the skin anti-aging effects.

## 2. Results and Discussion

### 2.1. Preparation and Characterization of Callus Extract from Citrus junos

Yellow *Citrus junos* callus synthesis was successfully induced after transferring the explants into Murashige and Skoog (MS) medium supplemented with 2.0 mg/L picloram, without generation of other plant growth regulators such as 2,4-dichlorophenoxyacetic acid (2,4-D) and 6-benzyladenine (BA). Plant cell proliferation at the point of explant injury may be due to the picloram accumulation at this point, which stimulates cell proliferation in the presence of growth regulators [20].

**Figure 1:**
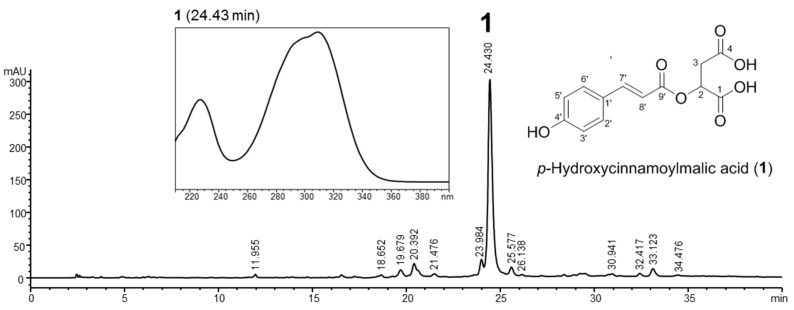
HPLC profile of the extract from *Citrus junos* callus and the structure of its major phenolic compound, *p*-hydroxycinnamoylmalic acid (**1**).

The chemical profile of *Citrus* callus extract was investigated using a high-performance liquid chromatograph with a photodiode array detector (HPLC-PDA). A major compound (**1**) with maximal absorption at 308 nm was detected, which corresponds to the UV spectrum of the *p*-hydroxycinnamoyl moiety (Figure 1). This compound was isolated by preparative HPLC, followed by spectroscopic analyses to elucidate its structure. Its NMR spectrum showed the presence of a *p*-hydroxycinnamoyl moiety composed of 1′,4′-disubstituted aromatic ring protons at δ 7.51 (2H, d, *J* = 8.4 Hz, H-2′,6′) and δ 6.90 (2H, d, *J* = 8.4 Hz, H-3′,5′), a *trans*-configuration double bond at δ 7.48 (1H, d, *J* = 15.9 Hz, H-7′) and δ 6.52 (1H, d, *J* = 15.9 Hz, H-8′), and a carbon ester at δ 168.6 (C-9′). The proton signals for hydroxymethine and methylene in a malic acid moiety were observed at δ 4.80 (1H, m, H-2) and δ 2.96 (2H, m, H-3) in the ^1^H-NMR spectrum, and carbon signals for two carboxylic acids were present at δ 175.0 and δ 174.8. Moreover, long-range ^1^H-^13^C heteronuclear correlations of H-2 to C-1 and C-9′ as well as H-3 to C-1 and C-4 were present in the HMBC spectrum. Based on these results and data reported previously [21], the main compound **1** in the callus extract was identified as *p*-hydroxycinnamoylmalic acid. The level of a major phenolic, *p*-hydroxycinnamoylmalic acid, in *Citrus junos* calli was subsequently quantified by HPLC using a solvent of acetonitrile and 0.1% formic acid aqueous solution, and a gradient of 5% to 40% acetonitrile over 40 min. A calibration curve for *p*-hydroxycinnamoylmalic acid was prepared at a concentration range of 10–1000 μg/mL, and recorded using peak areas (Y) and concentrations (X, μg/10 μL). The calibration curve exhibited good linear regression (Y = 421.78X − 33.43, *r*^2^ = 0.9997), and the *p*-hydroxycinnamoylmalic acid content in the callus extract of *Citrus junos* was determined to be 51.49 ± 0.01 mg/g. Minor LC peaks other than *p*-hydroxycinnamoylmalic acid were not identified in the callus extract due to the limited quantities available.

Cinnamic acids are aromatic carboxylic acids (C6–C3) that naturally occur in all green plants, being intermediates in the biosynthetic pathways leading to phenyl propanoids, flavonoids, isoflavonoids, coumarins, lignans, anthocyanins, spermidines, stilbenes, aurones, and tannins [22,23]. These secondary metabolites play essential physiological roles in plant development, growth, reproduction, and disease resistance [24,25]. The biological activities of phenolic cinnamic acids include antioxidant, anti-inflammatory, antitumor, and antimicrobial activities; multiple cytoprotective effects that ameliorate neuroinflammation in neurodegenerative diseases; anti-hypertensive activities; and anti-hyperlipidemic effects that minimize oxidation of low-density lipoprotein (LDL) [22,26,27,28,29]. In addition, phenolic acids can directly absorb and neutralize free radicals, and inhibit enzymes associated with ROS generation pathways (such as xanthine oxidase, NADPH oxidase, and myeloperoxidase) [29,30]. Phenolic acids also enhance the activities of human antioxidant enzymes, including superoxide dismutase and catalase [29,31].

The total phenol content of the extract was 24.50 ± 0.43 mg/g of gallic acid equivalents. However, flavonoids could not be detected by the corresponding AlCl_3_ method. Moreover, the HPLC-PDA profile of the callus extract did not exhibit an LC peak profile typical of *Citrus* flavanone glycosides, for example, hesperidin (UV λ_max_ 280 nm) and naringin (UV λ_max_ 282 nm), which are the major components of the fruits of *Citrus junos*. These results suggested remarkable differences in the major secondary metabolite composition of callus and plant extracts of *Citrus junos*.

**Figure 2:**
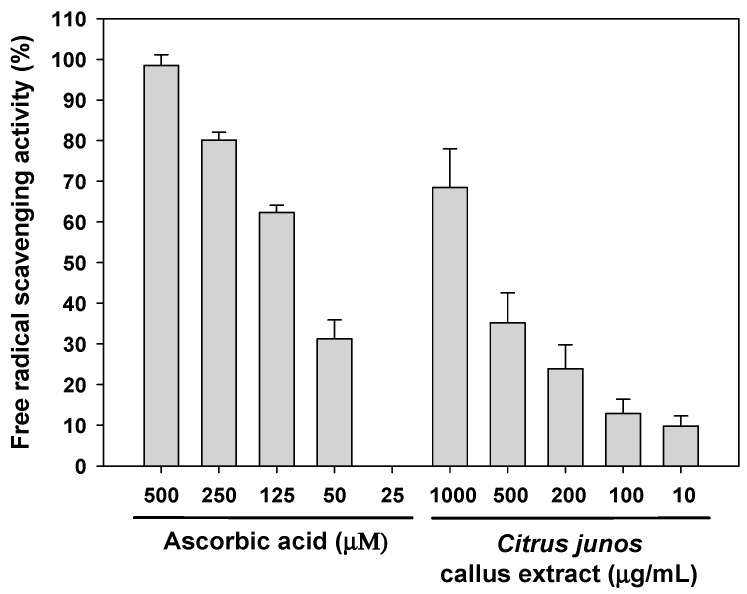
2,2-Diphenyl-2-picrylhydrazyl (DPPH) free radical-scavenging activity of *Citrus junos* callus extract. Values are mean ± standard deviation (*n* = 4 per group).

As a linear correlation between phenolic concentration and radical-scavenging activity has been reported in the extracts of many plants, vegetables, and fruits, we expected that the *Citrus junos* callus extract would exhibit strong antioxidant activity [30,31]. Figure 2 shows the DPPH radical-scavenging activity of the extract. Both ascorbic acid (positive control) and the extract exhibited dose-dependent radical-scavenging activity. At 1000 µg/mL, the extract exhibited 68.5 ± 9.49% free radical-scavenging activity, equivalent to that of 125 µM ascorbic acid (62.3 ± 1.78%), due principally to its phenolic components. However, the antioxidant activity of the callus extract was lower than that of ascorbic acid or cinnamic acid. Thus, further studies are required to optimize the culture conditions and extraction techniques to increase the functional compound content of the callus extract to enhance its antioxidant activity.

### 2.2. Inhibition of Tyrosinase Activity and Melanin Biosynthesis

**Figure 3:**
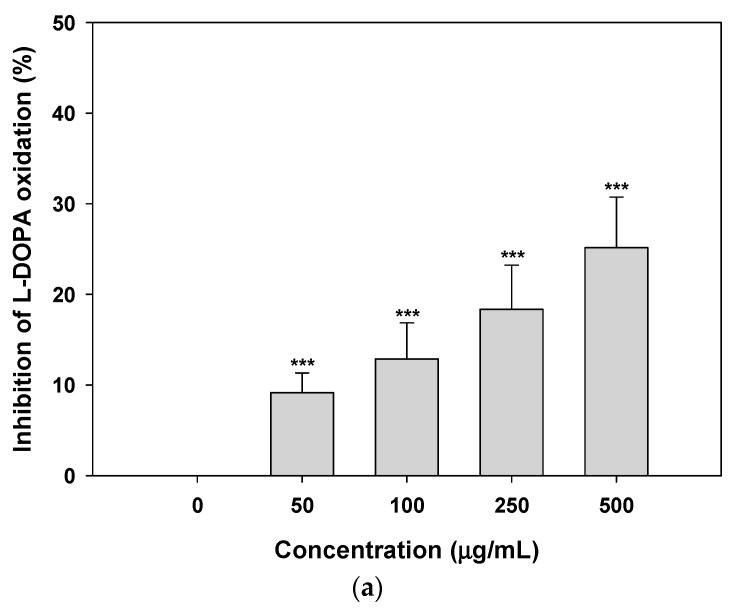

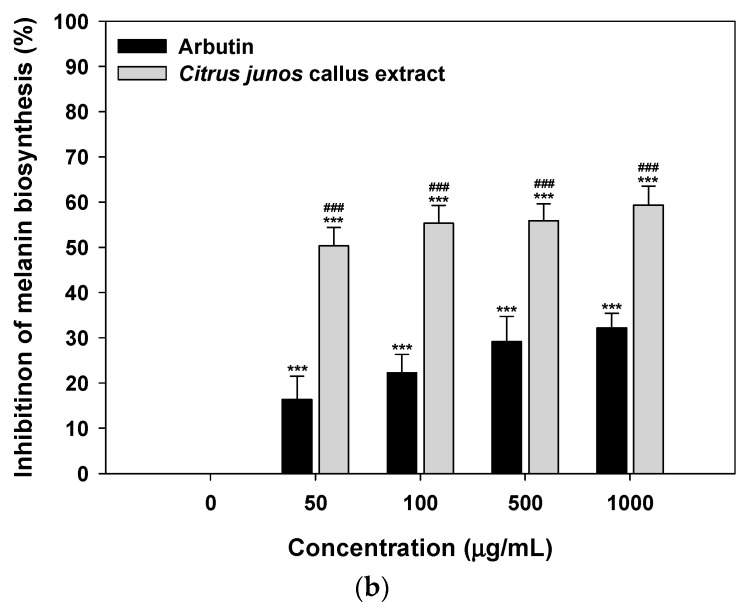
Inhibitory effects of *Citrus junos* callus extract on tyrosinase activity and melanin biosynthesis. (**a**) Relative mushroom tyrosinase activity assayed via l-3,4-dihydroxyphenylalanine (l-DOPA) oxidation in the absence or presence of the indicated concentrations of the extract; (**b**) melanin content of B16F10 melanoma cells. Data are mean ± standard deviation percentages of the control (*n* = 4 per group). *** *p* < 0.001 compared with the untreated control. ^###^ *p* < 0.001 compared with arbutin at the same concentration.

To assess the effects of *Citrus junos* callus extract on melanogenesis, we examined its ability to inhibit tyrosinase activity. As shown in Figure 3a, the extract inhibited l-3,4-dihydroxyphenylalanine (l-DOPA) oxidation in a dose-dependent manner; at 500 μg/mL, the magnitude of inhibition was 25.2 ± 5.57% that of the control. Moreover, the extract exerted a greater inhibitory effect on melanin synthesis in B16F10 melanoma cells than did arbutin at the same concentrations; inhibition of melanin synthesis in cells treated with 50 μg/mL extract was 208% that of cells treated with arbutin (16.3 ± 5.17%) (Figure 3b). Treatment with the extract at 1000 μg/mL resulted in maximum inhibition of melanin synthesis (59.3 ± 4.20%), which was 1.85-fold greater than that of arbutin (1000 μg/mL)-treated cells.

Lee et al. [32] reported that cinnamic acid and its derivatives inhibit tyrosinase activity because of their marked antioxidant effects; these materials consistently scavenge diverse ROS, including the hydroxyl radical, the peroxyl radical, the superoxide anion, hypochlorous acid, and peroxynitrite [33]. Kong et al. [34] also showed that cinnamic acid derivatives potently inhibit melanin production in melanocytes by reducing both tyrosinase activity and expression. This effect was associated with low-level cytotoxicity, resulting in depigmentation of UV-B-induced hyperpigmentation (brown guinea pig skin).

In this study, the callus extract at concentrations >5000 μg/mL inhibited tyrosinase activity; at 5000 μg/mL the extract inhibited tyrosinase activity by 23.1 ± 2.36% (35.5% lower than that by 5000 μg/mL arbutin [65.0 ± 5.03%]) (data not shown). However, the callus extract inhibited l-DOPA auto-oxidation in a dose-dependent manner and exerted a greater inhibitory effect on melanin biosynthesis than arbutin. Although further mechanistic studies are required, the anti-melanogenic effect of the callus extract may not be caused by direct inhibition of tyrosinase activity, such as copper chelation at the active site, but by inhibition of α-glucosidase and glycosylation of tyrosinase in melanoma cells [35,36].

### 2.3. Skin-Regeneration Activities of Citrus junos Callus Extract

#### 2.3.1. Effects of the Extract on Fibroblast Proliferation and Procollagen Synthesis

**Figure 4:**
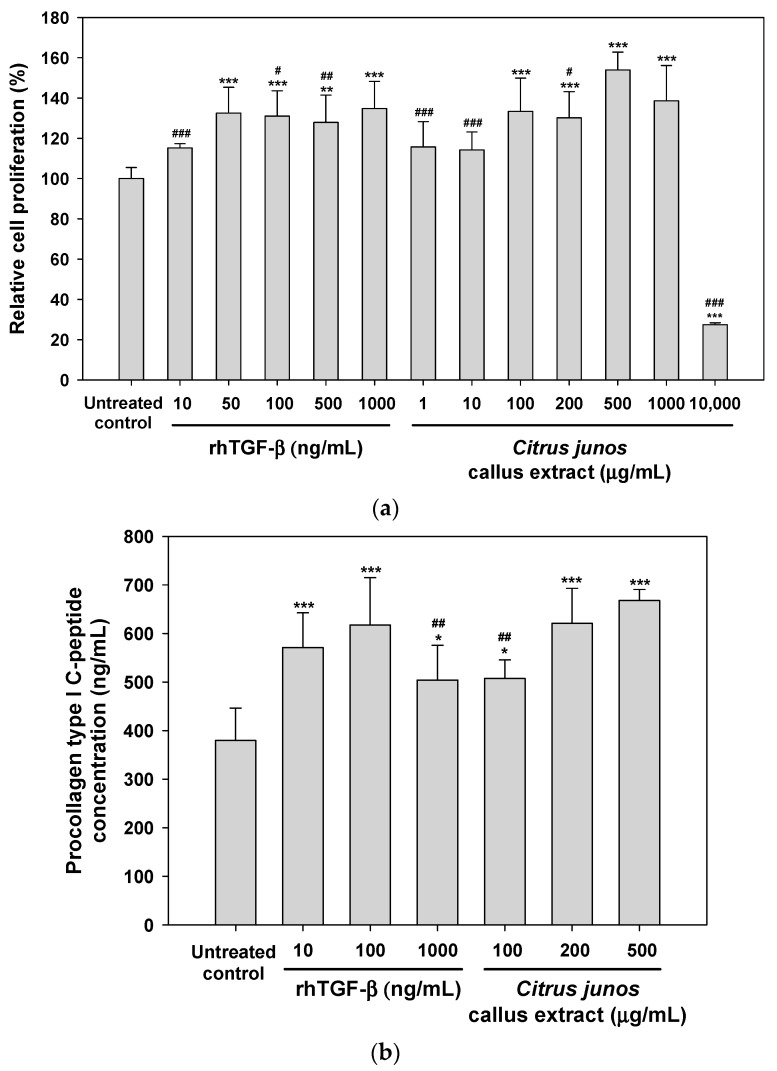
(**a**) Relative proliferation of and (**b**) procollagen type I C-peptide synthesis by CCD-986sk cells after treatment with recombinant human transforming growth factor-β (rhTGF-β) or *Citrus junos* callus extract for 24 h. Values are mean ± standard deviation (*n* = 6 per group). * *p* < 0.05, ** *p* < 0.01, *** *p* < 0.001 compared with the untreated control. ^#^ *p* < 0.05, ^##^ *p* < 0.01, ^###^ *p* < 0.001 compared with 500 μg/mL (final concentration) *Citrus junos* callus extract.

To evaluate the skin-regenerating activity of *Citrus junos* callus extract, we evaluated its effects on fibroblast proliferation. As shown in Figure 4a, human fibroblasts significantly proliferated after the addition of >50 ng/mL recombinant human transforming growth factor-β (rhTGF-β, positive control) and, at 500 ng/mL rhTGF-β, proliferation was 128 ± 13.6% greater than in the control. However, proliferation was not observed following treatment with <100 μg/mL callus extract. Maximum fibroblast proliferation with minimal toxicity was caused by treatment with 500 μg/mL callus extract (154 ± 8.88%). However, the extract at 10,000 μg/mL severely compromised fibroblast viability due to its cytotoxic effect. Thus, the extract at <1000 μg/mL is well tolerated and may not exert toxic effects on the skin.

Next, we examined the effect of the callus extract on collagen production by human fibroblasts. As shown in Figure 4b, the biosynthesis of procollagen type I C-peptide by fibroblasts was significantly enhanced by treatment with 10–1000 ng/mL rhTGF-β. Similarly, procollagen synthesis was promoted by the extract in a dose-dependent manner; the levels of synthesis of procollagen type I C-peptide after treatment with 100, 200 and 500 μg/mL (final concentrations) of the callus extract were 1.34-, 1.64-, and 1.76-fold greater than that of the control, respectively.

#### 2.3.2. In Vitro Scratch Wound Recovery Effect of *Citrus junos* Callus Extract

**Figure 5:**
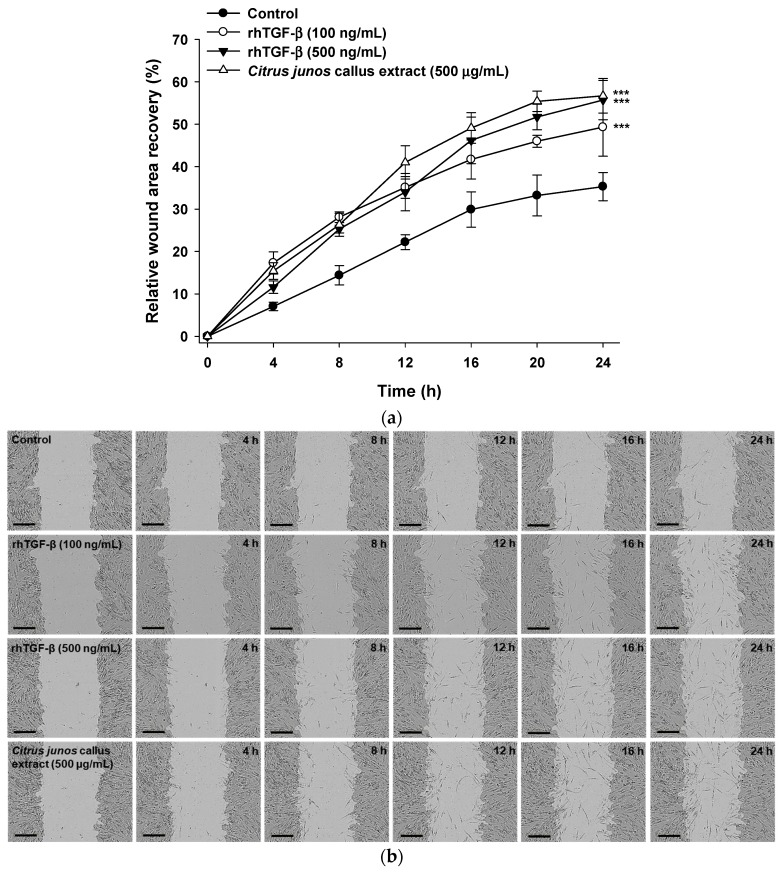
Relative scratch wound recovery of CCD-986sk cells after incubation with rhTGF-β or *Citrus junos* callus extract for 24 h. (**a**) Time-course of relative scratch wound recovery (area) of CCD-986sk cells; (**b**) representative micrographs of scratch wounds treated with rhTGF-β (100 and 500 ng/mL) or *Citrus junos* callus extract (500 μg/mL). Values are means ± standard deviation (*n* = 6 per group). *** *p* < 0.001 compared with the untreated control. Scale bar in (**b**): 300 µm.

Treatment with *Citrus junos* callus extract significantly accelerated wound closure compared with the control. An in vitro scratch wound recovery assay showed a 161% higher recovery rate at 24 h after treatment with 500 μg/mL *Citrus junos* callus extract compared with that in the control (Figure 5a). In addition, the callus extract (500 μg/mL final concentration) increased fibroblast proliferation and migration by 15% compared with rhTGF-β (100 ng/mL); the recovery rate was similar to that afforded by 500 ng/mL rhTGF-β (Figure 5b).

### 2.4. Characterization of NLs Containing Citrus junos Callus Extract

**Figure 6:**
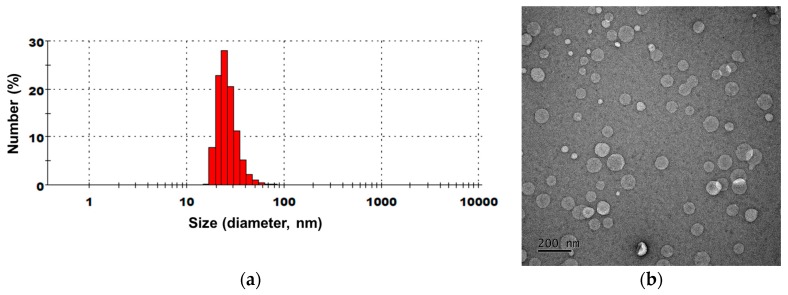
(**a**) Representative particle size distribution and (**b**) transmission electron micrograph of nanoliposomes containing *Citrus junos* callus extract (CC-NL#4). Scale bar in (**b**): 200 nm.

To improve the skin permeability of *Citrus junos* callus extract, extract-loaded NLs were prepared according to the formulations in Table 1. The average sizes, polydispersity indices (PDIs), and zeta potentials of liposomes produced by emulsification following microfluidization are summarized in Table 2. The liposomes exhibited ~80% mean encapsulation of the callus extract, and presented as nano-sized liposomes 44–147 nm in diameter with PDIs < 0.48. Except for CC-NL#2, all extract-loaded NLs had a highly negative surface charge, attributable to the incorporation of Lipoid P75-3. The mean particle size of CC-NL#4 was 146.8 ± 12.66 nm, with a narrow size distribution (PDI = 0.419). The zeta potential was −65.8 ± 2.29 mV. Transmission electron microscopy (TEM) revealed that all NLs were well-defined spheres of sizes similar to those determined by particle size analysis (Figure 6).

### 2.5. In Vitro Skin Permeability of Citrus junos Callus Extract-Loaded NLs

**Figure 7:**
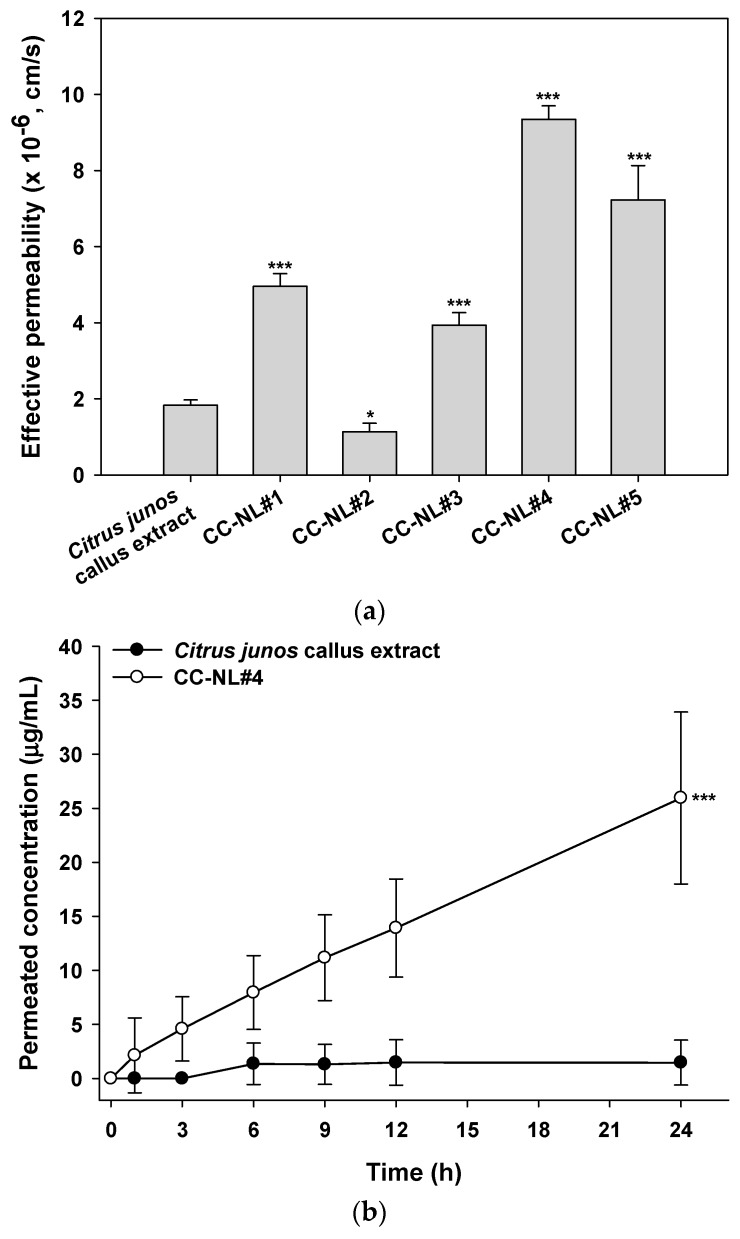
In vitro permeation of *Citrus junos* callus extract-loaded NLs across artificial skin (Skin PAMPA) and a human epidermal layer. (**a**) Effective permeability values of the callus extract or callus extract-loaded NLs through Skin PAMPA; (**b**) time-course of cumulative permeated concentrations of *p*-hydroxycinnamoylmalic acid from callus extract solution or callus extract-loaded NLs (CC-NL#4) through human epidermis layer. Values are mean ± standard deviation (*n* = 6 per group). * *p* < 0.05, *** *p* < 0.001 compared with the *Citrus junos* callus extract.

The artificial skin permeability of the Citrus junos callus extract was significantly increased by encapsulation into NLs, with the exception of formulation CC-NL#2 (Figure 7a). The skin permeability of the extract was influenced by the lipid composition of the NLs (Table 1 and Figure 7a). The permeabilities of NLs prepared using Lipoid P75-3 were higher than those of formulations containing Lipoid P100-3 (CC-NL#1 vs. CC-NL#2). In addition, the skin permeability of the callus extract increased as the cholesterol content of the NLs increased (CC-NL#1 vs. CC-NL#4). The maximum skin permeability of *p*-hydroxycinnamoylmalic acid from the extract-loaded NLs was from the formulation CC-NL#4, the permeability of which was 5.11-fold greater than that of the extract alone (9.34 ± 0.35 × 10^−6^ vs. 1.83 ± 0.14 × 10^−6^ cm/s).

In vitro skin permeation of *Citrus junos* callus extract across a human epidermal layer is shown in Figure 7b. The cumulative penetrated concentration of *p*-hydroxycinnamoylmalic acid from NLs of the CC-NL#4 formulation at 6 h was 5.84-fold greater than that of *Citrus junos* callus extract alone, resulting in a 17.67-fold increase in the skin flux of *p*-hydroxycinnamoylmalic acid after 24 h (6.89 ± 2.11 vs. 0.39 ± 0.55 μg/cm^2^/h).

Cholesterol enhances the rigidity and physical stability of liposomal membranes, thereby decreasing the leakage of liposome-loaded active compounds [37]. Furthermore, cholesterol increases the fluidity of phospholipids in the bilayer at temperatures lower than their melting point (*T*_m_), but decreases their fluidity if the bilayer is at temperatures above their *T*_m_ [38]. Therefore, the skin permeability of callus extract from NLs composed of hydrogenated phosphatidylcholine (HPC) and cholesterol may be enhanced because the *T*_m_ of phospholipids in the bilayer was lower than the skin temperature, creating a lipid-fluidizing effect and facilitating lipid incorporation into the intercellular domain and modification of skin lipids. Thus, *Citrus junos* callus extract-loaded NLs may be used as an anti-aging cosmetic that also exerts a skin-lightening effect.

## 3. Materials and Methods

### 3.1. Materials

Murashige and Skoog (MS) medium, picloram, 6-benzyladenine (BA), and 2,4-dichlorophenoxyacetic acid (2,4-D) were purchased from Duchefa Bohemia B.V. (Haarlem, The Netherlands). DPPH, ascorbic acid, l-tyrosine, l-3,4-dihydroxyphenylalanine (l-DOPA), tyrosinase, arbutin, and recombinant human transforming growth factor-β (rhTGF-β) were purchased from Sigma-Aldrich (St. Louis, MO, USA). Hydrogenated lecithin (Lipoid P75-3) and hydrogenated phosphatidylcholine (HPC; Lipoid P100-3) were obtained from Lipoid (Ludwigshafen, Germany). Other chemicals were purchased from Merck KGaA (Darmstadt, Germany) and Thermo Fisher Scientific Inc. (Waltham, MA, USA).

### 3.2. Preparation and Characterization of a Callus Extract of Citrus junos

#### 3.2.1. Preparation of Callus Extract

Leaves, flowers, and seeds of *Citrus junos* plants were washed with and soaked in water overnight, followed by immersion in 70% (*v*/*v*) ethanol for 90 s. Their surfaces were sterilized with 4% (*w*/*v*) aqueous solution of sodium hypochlorite for 15 min. After rinsing with sterile water four to five times, the explants were aseptically grown in MS basal medium (containing 3% sucrose and 0.26% gelrite [both *w*/*v*]) supplemented with various concentrations of plant growth regulators, including picloram, BA, or 2,4-D. To induce calli, the explants were injured with a 5 × 5-mm scalpel and inoculated onto freshly prepared medium, follow by maintenance in the dark for 1–8 weeks. After callus induction, calli were collected from the explants and transferred into fresh MS medium containing 2 mg/mL picloram, and sub-cultured every 2–3 weeks in a growth chamber at 26 ± 2 °C for a further 4 weeks. To increase the callus biomass, each callus was further cultured in MS liquid medium containing 3% (*w*/*v*) sucrose with 2 mg/L of picloram at 25 ± 1 °C, at 120 rpm, for 1–2 weeks. The calli were then harvested, air-dried at 50 °C, and pulverized using a mortar and pestle. To prepare an aqueous extract, the dried callus powder (200 g) was boiled in 1 L of distilled water for 4 h and filtered through a 0.4-μm pore size filter paper. Finally, the aqueous extract was freeze-dried to obtain a powder.

#### 3.2.2. Characterization of the Callus Extract by HPLC

To isolate the major chemical constituent, the dried aqueous extract (0.98 g) was subjected to preparative HPLC system (Waters 600 HPLC controller and pump with 2998 photodiode array detector) with Acclaim Polar Advantage II C18 (5 μm, 250 × 21.2 mm, Thermo Fisher Scientific Inc.), and separated using gradient solvent condition, composed of acetonitrile (A) and 0.1% formic acid-water (B), starting with 5% to 40% solvent A for 40 min at flow rate of 17.0 mL/min, to afford compound **1** (20 mg). 1D and 2D NMR data were obtained by Varian VNMRS600 spectrophotometer, and LC-MS were measured by Agilent 6120 quadruple LC/MS system at positive ESI mode.

*p*-Hydroxycinnamoylmalic acid (**1**): ^1^H-NMR (600 MHz, D_2_O): δ 7.51 (2H, d, *J* = 8.4 Hz, H-2′,6′), 7.48 (1H, d, *J* = 15.9 Hz, H-7′), 6.90 (2H, d, *J* = 8.4 Hz, H-3′,5′), 6.52 (1H, d, *J* = 15.9 Hz, H-8′), 4.80 (1H, m, H-2), 2.96 (2H, m, H-3); ^13^C-NMR (150 MHz, D_2_O): 175.0 (C-1), 174.8 (C-4), 168.6 (C-9′), 157.5 (C-4′), 141.7 (C-7′), 130.0 (C-2′,6′), 126.6 (C-1′), 116.7 (C-3′,5′), 115.7 (C-8′), 36.3 (C-3); ESI-MS 281.1 [M + H]^+^.

Quantification of *p*-hydroxycinnamoylmalic acid (**1**) in the callus extract was carried out using an Agilent 1260 Infinity HPLC system with a diode array detector using Acclaim Polar Advantage II C18 (5 μm, 250 × 4.6 mm, Thermo Fisher Scientific Inc.). The chromatographic condition was as follows: acetonitrile (A) and 0.1% formic acid-water (B), with 5% to 40% of solvent A for 40 min at flow rate of 1.0 mL/min under 320 nm.

#### 3.2.3. Determination of Antioxidant Capacity for the Callus Extract

The total phenolic content was assessed using the Folin-Ciocalteu reagent, as described by Singleton and Rossi [39]. Briefly, 1 mL of the callus extract was diluted with 9 mL water, and oxidized with 1 mL of diluted Folin-Ciocalteu reagent (Sigma-Aldrich) for 5 min at room temperature followed by neutralization with 10 mL sodium carbonate solution (7.0%, *w*/*v*). The mixture solution was diluted with water to final volume of 25 mL, and incubated for 90 min at room temperature. The absorbance at 750 nm was measured with a UV-VIS spectrophotometer (Biochrom Libra, Cambridge, UK). A blank sample of water in place of callus extract was used to distinguish any background absorbance. The total phenolic content was indicated as gallic acid equivalents (mg) per one gram of dry extract using a gallic acid calibration curve.

The total flavonoid content was estimated using the colorimetric method of Zhishen et al. [40]. Briefly, 1 mL of the callus extract was diluted to 5 mL with water and mixed with 0.3 mL of NaNO_2_ solution (5%, *w*/*v*). 0.3 mL of AlCl_3_ solution (10%, *w*/*v*) was added 5 min later, and 2 mL of NaOH solution (1 M) was treated, then made up to final volume of 10 mL with water. The absorbance of the mixture was determined spectrophotometrically at 510 nm (Biochrom Libra, Cambridge, UK). A standard curve that measured mg of catechin equivalents per one gram of dry extract was referenced to quantify total flavonoid content.

The efficacy of the callus extract as a radical scavenger was measured using the DPPH assay. DPPH (100 µL amounts of a 0.2 mM solution in methanol) was mixed with 100 µL amounts of ascorbic acid solutions or callus extract and the mixtures held in the dark for 30 min at room temperature. The negative and positive controls were prepared by addition of 100 µL amounts of DPPH solution to 100 µL methanol and callus extract, respectively. Then, absorbances of samples at 517 nm were measured against blank (100 µL DPPH solution + 100 µL water).

### 3.3. Skin-Lightening Activity

#### 3.3.1. Inhibition of l-DOPA Oxidation

To assess the inhibitory effects of *Citrus junos* callus extract on tyrosinase activity, 100 μL amounts of sample solution (50–500 µg/mL callus extract in 0.1 M phosphate-buffered saline (PBS, pH 6.8)) were mixed with 290 μL of 0.1 M PBS (pH 6.8) and 100 μL of 10 mM l-DOPA. After incubation at room temperature for 5 min, 10 μL of a mushroom tyrosinase solution (2500 units/mL in PBS [pH 6.8]) was added to the mixture and further incubated at 37 °C for 10 min. The absorbance was measured at 475 nm, and the inhibition of l-DOPA oxidation was calculated using the following equation:

Inhibition (%) = 100 − (B/A × 100)(1)

where A is the absorbance at 475 nm of the control, and B is the absorbance of the test sample at 475 nm.

#### 3.3.2. Inhibition of Melanin Biosynthesis in Melanocytes

To explore the inhibitory effect of callus extract on melanin synthesis in melanocytes, B16F10 melanoma cells (2 × 10^4^ cells/well) were seeded in a 24-well plate and stimulated with 100 nM α-melanocyte stimulating hormone (α-MSH) for 24 h. Next, the cells were treated with 50–1000 μg/mL (final concentration) of the callus extract or arbutin in 100 µL amounts of Dulbecco’s modified Eagle’s medium (DMEM) and incubated for a further 24 h. After washing twice with PBS (pH 7.4), the cells were detached by incubation in trypsin/ethylenediaminetetraacetic acid (EDTA). After centrifugation at 5000 rpm for 10 min, cell pellets were lysed using 150 µL amounts of 1 N NaOH at 65 °C for 45 min. The supernatants were harvested and melanin levels determined by measuring absorbance at 405 nm. The percentage inhibition of melanin synthesis was calculated by comparing the melanin levels in treated cells with those in untreated cells (100%).

### 3.4. Skin Regeneration Activity

#### 3.4.1. Fibroblast Proliferation Assay

To evaluate the effect of *Citrus junos* callus extract on human fibroblast (CCD-986sk) proliferation, 100 µL of cells in DMEM containing 10% (*v*/*v*) fetal bovine serum (FBS) and 1% (*w*/*v*) penicillin/streptomycin, were seeded in a 96-well plate at a density of 5 × 10^3^ cells/well. After 24 h incubation at 37 °C, the cells were cultured with DMEM with 0.05% (*v*/*v*) serum for an additional 24 h. Next, the callus extract or rhTGF-β in DMEM with 0.5% (*v*/*v*) FBS was added to the cells. After 24 h of incubation, cells were incubated with 5 mg/mL of WST-1 in PBS (pH 7.4) (Roche Diagnostics, Mannheim, Germany) for 2 h. The absorbance at 450 nm was then measured and the percentage of viable cells was determined in comparison to the absorbance of untreated cells.

#### 3.4.2. Procollagen Synthesis Assay

To evaluate whether callus extract exhibited anti-wrinkle activity by promotion of collagen synthesis, the level of procollagen type I C-peptide produced by fibroblasts was measured as a marker for procollagen synthesis. CCD-986sk cells were seeded and cultured in a 96-well plate at a density of 1 × 10^4^ cells/well in 100 µL amounts of DMEM with 10% (*v*/*v*) FBS until the cells reached 80% confluency. After overnight incubation in DMEM containing 0.5% (*v*/*v*) FBS, the cells were treated with various concentrations of rhTGF-β or the callus extract in serum-free medium and incubated for 24 h. Then, the supernatants of the culture medium were harvested and the concentrations of procollagen type I C-peptide were measured using an enzyme-linked immunosorbent assay (ELISA) kit (Takara Bio, Shiga, Japan) in accordance with the manufacturer’s instructions.

#### 3.4.3. In Vitro Scratch Wound Recovery Assay

To explore the skin regenerative activities of *Citrus junos* callus extract, CCD-986sk cells were seeded in a 96-well plate at a density of 1 × 10^4^ cells/well and cultured to form a confluent monolayer. Then, a cell-free zone (scratch) was created in each well using a wound-maker. After washing twice with PBS (pH 7.4), the cells were cultured in serum-free medium at 37 °C in the presence of 100 and 500 ng/mL of rhTGF-β, or 500 μg/mL of the callus extract. Wound closure was monitored every 4 h using an Incucyte Zoom microscope equipped with image analysis software (Essen Bioscience Inc., Ann Arbor, MI, USA) to calculate cell recovery areas via phase-contrast imaging.

### 3.5. Preparation and Characterization of Citrus junos Callus Extract-Loaded NLs

*Citrus junos* callus extract-loaded NLs were prepared via emulsification followed by microfluidization. Briefly, Lipoid P75-3, Lipoid P100-3, cholesterol, caprylic capryl triglyceride, and ethanol were dissolved in glycerol at 60 °C to form an oil. Separately, the callus extract was dissolved in water (an aqueous solution). The compositions of all NLs are listed in Table 1. The oil was slowly added to the aqueous solution and mixed at 3000 rpm using a homogenizer (Primix Corp., Hyogo, Japan) to produce the primary emulsion. After mixing for 10 min, the emulsion was passed through a microfluidizer (LM20; Microfluidics Corp., Westwood, MA, USA) operating at 1000 bar (10 cycles) to form NLs.

To determine the average encapsulation efficiency of the callus extract in the NLs, 0.5 mL of the dispersed NLs was centrifuged at 18,000 rpm for 1 h. Next, the concentration of unincorporated *p*-hydroxycinnamoylmalic acid in the supernatant was determined by HPLC at 320 nm, as described above. Furthermore, dynamic light scattering (Malvern Zetasizer Nano ZS90; Malvern Instruments, Malvern, UK) was used to characterize the average hydrodynamic diameters, PDIs, and zeta potentials of the NLs at 25 °C after dilution in deionized water (1:20). For morphological evaluation, NLs were further diluted × 100 in deionized water, with drops placed on copper grids. After removal of excess liquid with filter paper, a drop of 2% (*w*/*v*) aqueous phosphotungstic acid was added for negative staining and the grid examined by TEM (JEM-200; JEOL, Tokyo, Japan).

### 3.6. In Vitro Skin Permeability of Citrus junos Callus Extract-Loaded NLs

#### 3.6.1. Permeation across an Artificial Skin

The in vitro skin permeabilities of the callus extract solution or callus extract-loaded NLs across artificial skin were assessed using the Skin PAMPA Explorer Test System (Pion Inc., Billerica, MA, USA). After overnight hydration of each well of the top (donor) compartment with 200 μL diluted hydration solution (pH 7.4), each well of the bottom (acceptor) compartment was filled with 200 μL Prisma-HT buffer solution (pH 7.4). Then, 200 μL amounts of callus extract or callus extract-loaded NLs diluted with Prisma-HT buffer were loaded into donor wells. The resulting Skin PAMPA sandwich was incubated at room temperature, and 150 μL amounts of the donor and acceptor solutions of the sandwiches were collected at 6, 12 and 24 h after loading. The levels of *p*-hydroxycinnamoylmalic acid in the samples were quantified by HPLC by measuring absorbance at 320 nm, as described above.

#### 3.6.2. In Vitro Permeability through a Human Epidermal Layer

To evaluate the permeability of callus extract or a callus extract-loaded NL (CC-NL#4) through an epidermal layer, excised human epidermis (Huskin; HansBiomed Corp., Daejeon, Korea) was mounted with the SC facing upward on the receptor compartment of a Franz diffusion cell system filled with 5 mL PBS (pH 7.4). The donor compartment was then clamped in place (the diffusion area was 0.785 cm^2^). After equilibrium was attained, 500 μL amounts of callus extract or CC-NL#4 diluted in PBS (pH 7.4) (equivalent to 500 μg/mL *p*-hydroxycinnamoylmalic acid) were added to the donor compartments. The receptor compartments were stirred at 600 rpm and a heating system was used to maintain a skin surface temperature of 32 °C throughout the experiment. After 1, 3, 6, 9, 12 and 24 h, 200 μL amounts of the receptor phase were withdrawn and replaced with fresh PBS (pH 7.4). The samples were filtered through polyvinylidene fluoride membrane filters (0.45 μm in pore size) and permeated *p*-hydroxycinnamoylmalic acid from the callus extract or the callus extract-loaded NL was quantified via HPLC by measuring absorbance at 320 nm, as previously described.

### 3.7. Statistics

All data are expressed as means ± standard deviation. A *p*-value <0.05 was considered to reflect statistical significance when the *t*-test was used to compare two mean values of unpaired data, or on one-way analysis of variance (ANOVA) followed by Tukey’s multiple comparison test when more than three mean values of unpaired data were compared.

## 4. Conclusions

In this work, *Citrus junos* plant callus was produced by transferring the explants into MS medium supplemented with picloram as a growth regulator. The total phenol content in callus extracts was 24.50 ± 0.43 mg/g, and the level of *p*-hydroxycinnamoylmalic acid, the major phenolic compound was found as 51.49 ± 0.01 mg/g in the extract. The DPPH free radical-scavenging efficacy of the callus extract (1000 μg/mL) was equivalent to that of 125 µM ascorbic acid. The callus extract also exhibited anti-tyrosinase activity, and both l-DOPA oxidation and melanin biosynthesis were significantly reduced by treatment with the extract at >50 μg/mL. Fibroblasts proliferation and their procollagen synthesis were significantly promoted (by 154% and 176%, respectively) after treatment with 500 μg/mL of callus extract. In addition, the callus extract significantly accelerated scratch wound recovery in vitro compared with the control. The flux of *Citrus junos* callus extract incorporated into NLs through the human epidermis was 17.67-fold greater than that of the callus extract alone. Thus, *Citrus junos* callus extract-loaded NLs may serve as an anti-aging cosmeceutic with a skin-lightening effect.

## Figures and Tables

**Table 1:** Formulations of nanoliposomes containing *Citrus junos* callus extract.

Component (%)	CC-NL#1	CC-NL#2	CC-NL#3	CC-NL#4	CC-NL#5
Lipoid P75-3	5	0	2.5	5	2.5
Lipoid P100-3	0	5	2.5	0	2.5
Cholesterol	0	0	0	2	1
Squalene	1	1	1	0	1
Caprylic capryl triglyceride	0	0	0	0	5
Ethanol	1	1	1	1	1
Glycerol	50	50	50	50	50
Citrus callus extract	10	10	10	10	10
Water	33	33	33	32	27

**Table 2:** Mean particle sizes, polydispersity indices, and zeta potentials of *Citrus junos* callus extract-loaded nanoliposomes.

Formulation Code	Particle Size (nm)	Polydispersity Index	Zeta Potential (mV)
CC-NL#1	44.12 ± 0.889	0.482 ± 0.026	−63.4 ± 0.55
CC-NL#2	51.04 ± 0.404	0.083 ± 0.001	0.59 ± 0.08
CC-NL#3	122.3 ± 1.818	0.285 ± 0.015	−48.1 ± 0.85
CC-NL#4	146.8 ± 12.66	0.419 ± 0.062	−65.8 ± 2.29
CC-NL#5	73.15 ± 1.226	0.280 ± 0.006	−54.5 ± 3.10
